# 

**Published:** 1881-12

**Authors:** 


					﻿ESTABLISHED IN 1855,
OldPeeie®.	BTCEM RT”R. lPfil	New Sei ies.
Vol.	9.	UXV^LUDXIV, 1CO1.	Vol. 1-No 3.
ATLANTA (
MEDICAL REGISTER.
{ATLANTA MEDICAL AND SURGICAL JOURNAL.)
EZITCBS:
JNO. TIIAD. JOHNSON, M.D., JAS- B. BAIRD, M.D.
PUBLISHED BY H. H. DICKSON, AT $2.50 A YE/R’IN ADVANCE.
>
X
ATLANTA, GEORGIA:
H. 11. DICKSOB, BOOK ABD JOB 1’RIBIER AB D BGGKB1BDER
1881.
				

